# Stage 4 s neuroblastoma: features, management and outcome of 268 cases from the Italian Neuroblastoma Registry

**DOI:** 10.1186/s13052-018-0599-1

**Published:** 2019-01-11

**Authors:** Bruno De Bernardi, Andrea Di Cataldo, Alberto Garaventa, Paolo Massirio, Elisabetta Viscardi, Marta Giorgia Podda, Aurora Castellano, Paolo D’Angelo, Elisa Tirtei, Fraia Melchionda, Simona Vetrella, Francesco De Leonardis, Carmelita D’Ippolito, Annalisa Tondo, Antonella Nonnis, Giovanni Erminio, Anna Rita Gigliotti, Katia Mazzocco, Riccardo Haupt

**Affiliations:** 1 Department of Hematology-Oncology, IRCCS Istituto Giannina Gaslini, Via Gaslini 5, 16147 Genoa, Italy; 2 Department of Pediatric Hematology-Oncology, University Hospital, Catania, Italy; 3 Department of Pediatrics, University Hospital, Padova, Italy; 4 Department of Oncology, Istituto Nazionale Tumori, Milan, Italy; 5 Department of Pediatric Oncology, Bambino Gesù Children’s Hospital, Rome, Italy; 6 Department of Pediatrics, University of Palermo, Palermo, Italy; 7 Department of Pediatric Hematology-Oncology, Regina Margherita Hospital, Torino, Italy; 8 Hematology-Oncology Unit, Sant’Orsola-Malpighi Policlinic, Bologna, Italy; 9 Department of Hematology-Oncology, Santobono-Pausilipon Children’s Hospital, Naples, Italy; 10 Department of Pediatrics, University Hospital, Bari, Italy; 11 Department of Pediatrics, Civic Hospital, Brescia, Italy; 12 Department of Hematology-Oncology, Anna Meyer Children’s Hospital, Florence, Italy; 13 Pediatric Onco-Hematology, Civic Hospital, Cagliari, Italy; 14 Epidemiology and Biostatistics Unit, IRCCS Istituto Giannina Gaslini, Genoa, Italy; 15 Pathology Unit, IRCCS Istituto Giannina Gaslini, Genoa, Italy

**Keywords:** Neuroblastoma, Infants, Stage 4 s, Prognostic factors

## Abstract

**Background:**

Infants diagnosed with stage 4 s neuroblastoma commonly experience spontaneous disease regression, with few succumbing without response to therapy. We analyzed a large cohort of such infants enrolled in the Italian Neuroblastoma Registry to detect changes over time in presenting features, treatment and outcome.

**Methods:**

Of 3355 subjects aged 0–18 years with previously untreated neuroblastoma diagnosed between 1979 and 2013, a total of 280 infants (8.3%) had stage 4 s characteristics, 268 of whom were eligible for analyses. Three treatment eras were identified on the basis of based diagnostic and chemotherapy adopted. Group 1 patients received upfront chemotherapy; Group 2 and 3 patients underwent observation in the absence of life-threatening symptoms (LTS), except for Group 3 patients with amplified *MYCN* gene, who received more aggressive therapy.

**Results:**

The three groups were comparable, with few exceptions. Ten-year overall survival significantly increased from 76.9 to 89.7% and was worse for male gender, age 0–29 days and presence of selected LTS on diagnosis, elevated LDH, and abnormal biologic features. Infants who underwent primary resection ± chemotherapy did significantly better. On multivariate analysis, treatment eras and the association of hepatomegaly to dyspnea were independently associated with worse outcome.

**Conclusions:**

Our data confirm that stage 4 s neuroblastoma is curable in nearly 90% of cases. Hepatomegaly associated to dyspnea was the most important independent risk factor. The cure rate could be further increased through timely identification of patients at risk who might benefit from surgical techniques, such as intra-arterial chemoembolization and/or liver transplantation, which must be carried out in institutions with specific expertise.

**Electronic supplementary material:**

The online version of this article (10.1186/s13052-018-0599-1) contains supplementary material, which is available to authorized users.

## Background

The intriguing subset of neuroblastoma named stage IV-S was described by D’Angio et al. in 1971 and referred to patients who would otherwise be stage I or II, but who had remote disease confined only to one or more of the following sites: liver, skin, or bone marrow [1]. Subsequently the International Neuroblastoma Staging System (INSS) introduced the age limit of 1 year and the degree of bone marrow infiltration less than 10% and reclassified such cases as stage 4 s [2]. Finally, in 2008, the International Neuroblastoma Risk Group Staging System (INRGSS) raised the patient age limit to 18 months [3]. The typical natural history of stage 4 s is characterized by an initial phase of tumor progression lasting a variable number of days/months, usually followed by spontaneous regression, the mechanism of which has not yet been clarified. [4] In a minority of patients, however, stage 4 s disease progresses independently of any therapy, leading to death. This outcome has been recorded in several studies, which have reported survival rates ranging from 60 to 90% [5–14].The outcome of infants diagnosed with stage 4 s disease has been seen to be negatively affected by several factors: age < 2 months [9, 11], life-threatening symptoms (LTS) [15, 16] and some biologic features of tumor cells [11, 13, 17–22]. However, several issues remain poorly defined, i.e.*,* which patients may benefit from chemotherapy, the timing and effect of primary tumor resection, the management of patients with unfavorable biologic features, and the feasibility and benefit of some surgical procedures in the case of massive liver enlargement.

In this study, we aimed to describe the modifications in presenting features and survival probabilities that occurred over a 34-year period in a cohort of stage 4 s infants enrolled in the RINB [23], and to define the impact of presenting features on patient outcome.

## Methods

Between January 1979 and December 2013, a total of 3355 subjects aged 0–18 years with previously untreated neuroblastoma were diagnosed in 27 AIEOP (Italian Association of Pediatric Hematology-Oncology) institutions and registered in the RINB. Of these, 280 (8.3%) had stage 4 s characteristics and were eligible for this study. Patients’ clinical records were reviewed to obtain details regarding the LTS arbitrarily defined as “major symptoms”: *i)* hepatomegaly, *ii)* dyspnea, and *iii)* organ dysfunctions.

### Diagnosis and diagnostic work-up

Tumor diagnosis was based on the combination of clinical and biochemical data and adequate imaging. After 1985, the diagnosis was usually confirmed by histopathology. From 2000 onwards, histology was centrally reviewed on the basis of the INPC (International Neuroblastoma Pathology Classification) criteria [24]. The diagnostic work-up included imaging studies, local assay of urinary catecholamine metabolites, LDH and ferritin serum levels, and at least one bone marrow aspirate. After 1985, tumor specimens were evaluated for biologic features at a single reference laboratory.

### Treatment

All patients received supportive care. Early resection of the primary tumor was encouraged, while late resection of a a residual mass after tumor shrinkage was based on institutional decision. In this study, the term resection refers to radical resection of the primary tumor [25, 26]. Liver irradiation usually consisted of a total dose of 1.5 Gy divided over 3 consecutive days. The chemotherapeutic approach varied during the study period. Three treatment eras were identified (Additional file 1: Table S1). In the first era (1979–84), chemotherapy was administered independently of clinical presentation. In the second era (1985–1999), treatment was based on symptoms on presentation: in children without LTS a wait-and-see policy was encouraged, while patients with LTS received 2–4 courses of various drug associations. In the third era (2000–2013), patients were treated in accordance with the therapeutic guidelines of an ad hoc SIOPEN (International Society of Pediatric Oncology Europe Neuroblastoma) protocol; those with amplified *MYCN* were candidates for an intensive therapeutic approach [27].

### Statistical analyses

Descriptive statistics are reported as absolute frequencies and percentages for qualitative variables, and as median values with their related interquartile range (IQR) for quantitative variables. To compare proportions between groups, Pearson’s chi-square and Fisher’s exact test, when appropriate, were applied. In the univariate analysis of each risk factor, progression-free survival (PFS) and overall survival (OS) were estimated by means of the Kaplan-Meier method, and differences between groups were assessed by means of the log-rank test. Survival estimates referred to the 10 years following diagnosis, and the related 95% confidence intervals (95%CI) were obtained by means of the Kalbfleisch and Prentice method. Finally, a multivariable Cox regression model was fitted in order to evaluate the combined effect of variables. In this analysis, only variables found to significantly affect PFS or OS were included in the model. All tests were two-tailed and a *P* value <.05 was considered statistically significant. All analyses were performed by means of Stata Statistical Software (Release 13.1, Stata Corporation, College Station, TX, USA).

## Results

On reviewing the records of the 280 stage 4 s infants enrolled in the RINB, 12 were excluded because of insufficient data (*n* = 10) or unconfirmed stage (*n* = 2). Of the 268 patients evaluable for analyses, 26 were enrolled in the first, 116 in the second, and 126 in the third era (accounting for 7.2, 9.2 and 7.3% of patients diagnosed in the respective periods).

### Demographic and clinical features on presentation

**Fig. 1:**
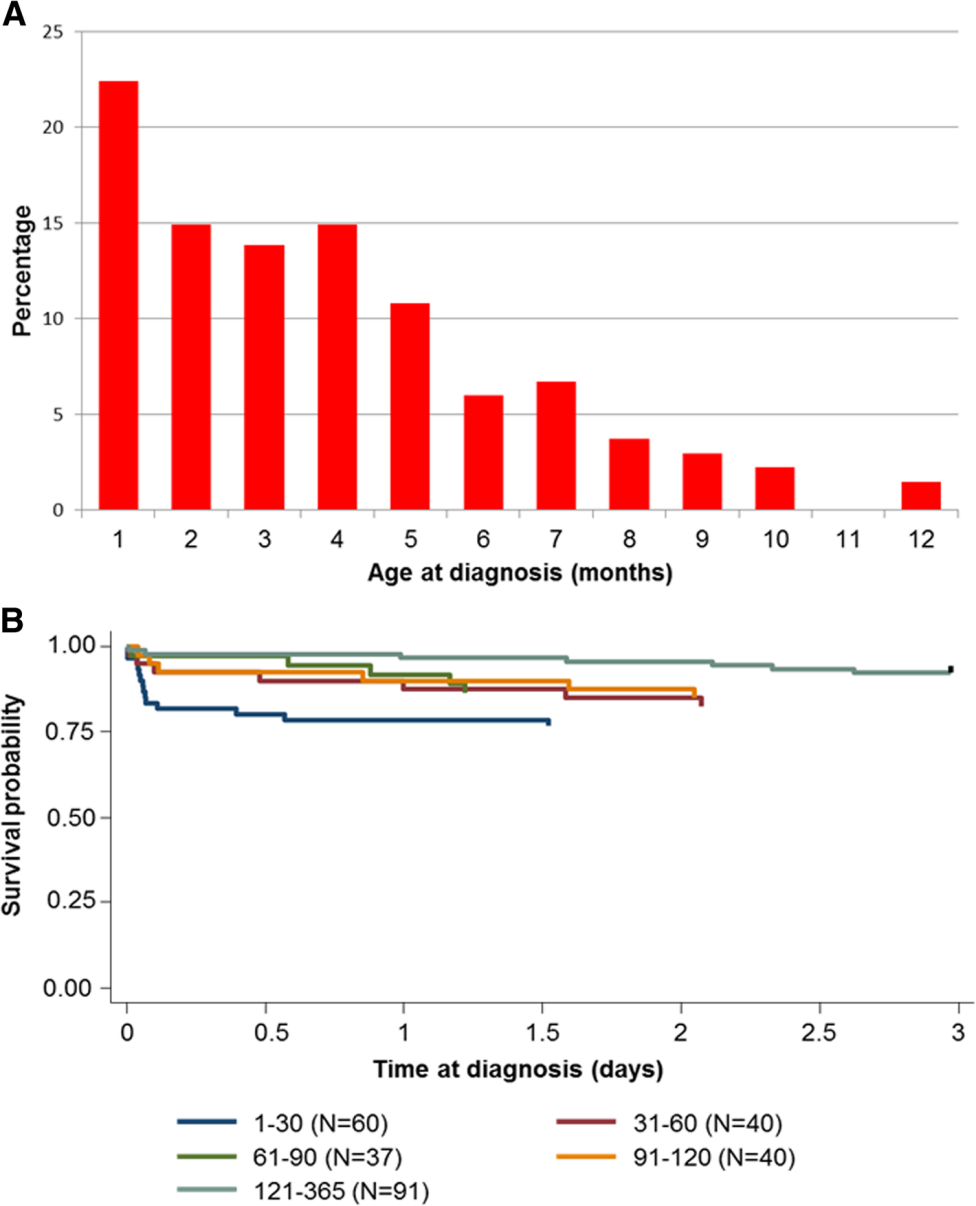
Incidence by age on diagnosis (**a**) and 3-year OS (**b**) in 268 stage 4 s neuroblastoma patients

Patient features in the entire cohort and during the 3 eras are listed in Table 1. The prevalence of the main features in the three treatment eras were roughly comparable. Male sex prevailed (58.2%). Median age on diagnosis was 3 months (IQR range, 2–5) with 22.4% of patients diagnosed in the first month of life, followed by a gradual decrease (Fig. 1 plot A).

**Table 1:** Presenting features of 268 stage 4 s neuroblastoma patients

Feature	All patients	Treatment era			*p*
		1979–1984	1985–1999	2000–2013	
	No. (%)	No. (%)	No. (%)	No. (%)	
	268 (100)	26 (9.7)	116 (43.3)	126 (47.0)	
**Demographic and clinical features**					
Gender					
Male	156 (58.2)	17 (65.4)	70 (60.3)	69 (54.8)	*0.0501*
Female	112 (41.8)	9 (34.6)	46 (39.7)	57 (45.2)	
Age, days. Median (IQR)	87 (33.5–146.5)	75.5 (34–122)	77 (22.5–141.5)	97.5 (37–170)	
Age, days					
0–29	60 (22.4)	6 (23.1)	31 (26.7)	23 (18.3)	*0.619*
30–59	40 (14.9)	4 (15.4)	14 (12.1)	22 (17.5)	
60–89	37 (13.8)	6 (23.1)	15 (12.9)	16 (12.7)	
90–119	40 (14.9)	3 (11.5)	19 (16.4)	18 (14.3)	
120–365	91 (33.9)	7 (26.9)	37 (31.9)	47 (37.3)	
Age, days					
0–29	60 (22.4)	6 (23.1)	31 (26.7)	23 (18.3)	*0.286*
30–365	208 (77.6)	20 (76.9)	85 (73.3)	103 (81.7)	
Symptoms at presentation^#^					
None	17 (6.3)	0	4 (3.5)	13 (10.3)	0.039*
Yes, minor	51 (19.0)	3 (11.5)	19 (16.4)	29 (23.0)	
Yes, major	200 (74.6)	23 (88.5)	93 (80.2)	84 (66.7)	
Major symptoms	200 (74.6)	23 (88.5)	93 (80.2)	84 (66.7)	
Hepatomegaly, yes	186 (69.4)	21 (80.8)	85 (73.3)	80 (63.5)	*0.107*
Dyspnea, yes	52 (19.4)	4 (15.4)	25 (21.5)	23 (18.2)	*0.699*
Organ dysfunction, yes	34 (12.7)	3 (11.5)	9 (7.8)	22 (17.5)	0.073*
Combinations of major symptoms					
No major symptoms or no symptom	68 (25.4)	3 (11.5)	23 (19.8)	42 (33.3)	0.033*
Organ dysfunction only	2 (0.8)	1 (3.9)	0	1 (0.8)	
Dyspnea ± Organ dysfunction	12 (4.5)	1 (3.9)	8 (6.9)	3 (2.4)	
Hepatomegaly ± Organ dysfunction	146 (54.5)	18 (69.2)	68 (58.6)	60 (47.6)	
Hepatomegaly + Dyspnea (± Organ dysfunction)	40 (14.9)	3 (11.5)	17 (14.7)	20 (15.9)	
Minor symptoms					
Skin nodules, yes	42 (15.7)	4 (15.4)	28 (24.1)	10 (7.9)	0.002*
Abdominal mass, yes	34 (12.7)	1 (3.8)	12 (10.3)	21 (16.7)	0.139*
Cervical mass, yes	11 (4.1)	0	5 (4.3)	6 (4.8)	0.814*
Neurologic symptoms, yes	12 (4.5)	0	6 (5.2)	6 (4.8)	0.753*
Primary site					
Adrenal^	175 (65.3)	16 (61.5)	77 (66.4)	82 (65.1)	0.272*
Retroperitoneal ganglia	49 (18.3)	4 (15.4)	16 (13.8)	29 (23.0)	
Thorax	22 (8.2)	3 (11.5)	11 (9.5)	8 (6.3)	
Neck	9 (3.4)	0	5 (4.3)	4 (3.2)	
Not identified	13 (4.9)	3 (11.5)	7 (6.0)	3 (2.4)	
Liver infiltration, yes					
yes	230 (85.8)	22 (84.6)	102 (87.9)	106 (84.1)	0.689*
Positive bone marrow cytology, yes					
yes	111 (41.4)	8 (30.8)	42 (36.2)	61 (48.4)	0.082*
**Biochemical, biologic and histologic features**					
Urine VMA (222 tested)					
Normal	59 (26.6)	2 (8.3)	22 (22.9)	35 (34.3)	0.019
Elevated	163 (73.4)	22 (91.7)	74 (77.1)	67 (65.7)	
Urine HVA (112 tested)					
Normal	18 (16.1)	0	2 (6.1)	16 (21.0)	0.149*
Elevated	94 (83.9)	3 (100)	31 (93.9)	60 (79.0)	
Serum LDH (227 tested)					
Normal	131 (57.7)	7 (87.5)	78 (75.7)	46 (39.7)	< 0.001*
Elevated	96 (42.3)	1 (12.5)	25 (24.3)	70 (60.3)	
Serum ferritin (193 tested)					
Normal	116 (60.1)	5 (100)	55 (61.1)	56 (57.1)	0.176*
Elevated	77 (39.9)	0	35 (38.9)	42 (42.9)	
*MYCN* gene (183 tested)					
Normal	168 (91.8)	0	61 (91.0)	107 (92.2)	0.776
Amplified	15 (8.2)	0	6 (9.0)	9 (7.8)	
1p chromosome (138 tested)					
Normal	110 (79.7)	1 (100)	32 (76.2)	77 (81.1)	0.603*
Deleted	28 (20.3)	0	10 (23.8)	18 (18.9)	
DNA index (121 tested)					
Aneuploid	80 (66.1)	0	24 (60.0)	56 (69.1)	0.318
Di-tetraploid	41 (33.9)	0	16 (40.0)	25 (30.9)	
Histology by INPC (75 tested)					
Favorable	69 (92.0)	0	0	69 (92.0)	–
Unfavorable	6 (8.0)	0	0	6 (8.0)	

*Abbreviations. IQR interquartile range, VMA vanillylmandelic acid, HVA homovanillic acid, LDH lactate dehydrogenase, INPC International Neuroblastoma Pathology Classification*

*#, patients may have more than one symptom*

* Fisher exact test

*^, 9 (3.4%) bilateral adrenal primary*

Seventeen patients (6.3%) were asymptomatic, as the tumor was detected by ultrasound examination performed in late pregnancy (*n* = 2) or during post-natal screening (*n* = 15). This occurred in an increasing number of patients over the 3 eras (0, 3.5, and 10.3%, respectively; *P* = .039) (Table 1). Two hundred patients (74.6%) presented with at least one major symptom, the most frequent being hepatomegaly (*n* = 186; 69.4%), with decreasing incidence over the 3 eras (not significant), followed by dyspnea (*n* = 52; 19.4%), and at least one organ dysfunction (*n* = 34;12.7%). (Table 1).

Other symptoms were: *i)* skin nodules (42 patients = 15.7%), with different incidence in the 3 eras (15.4% vs. 24.1% vs. 7.9%; *P* = .002); *ii)* abdominal mass (in the absence of hepatomegaly) (34 patients = 12.7%), with decreasing incidence over the study period (not significant); *iii)* cervical mass (11 patients = 4.1%); and *iv)* neurological abnormalities (12 patients = 4.5%) (Table 1).

The primary tumor site was most often identified in the adrenal (*n* = 175; 65.3%), including 9 bilateral cases (3.4%), followed by retroperitoneal ganglia (*n* = 49; 18.3%), thorax (*n* = 22; 8.2%), and neck (*n* = 9; 3.4%). In 13 patients (4.9%), a primary tumor was not identified. Hepatic involvement was documented in 230 patients (85.8%). Bone marrow infiltration was detected on light microscopy examination in 111 patients (41.4%) (Table 1).

### Biochemical, biologic and histopathologic data

Vanillylmandelic acid (VMA) urinary excretion was found elevated in 163 of 222 patients tested (73.4%); the number of cases with abnormal values decreased significantly over the 3 eras (*P* = .019). Homovanillic acid (HVA) excretion was found elevated in 94 of 112 patients tested (83.9%). The serum level of LDH was found elevated in 96 out of 227 patients (42.3%), with significant differences among the 3 groups (12.5% vs. 24.3% vs. 60.3%) (*P* < .001). Serum ferritin was found elevated in 77 out of 193 patients (39.9%).

Biologic features were evaluated in patients in the second and third eras only. *MYCN* gene was amplified in 15 out of 183 tumors (8.2%). Chromosome 1p was found deleted in 28 of 138 tested tumors (20.3%) and DNA index was di- or tetraploid in 41 of 121 tumors tested (33.9%). In the third era, histopathology of 75 tumors was centrally evaluated with 69 being rated favorable (92.0%) (Table 1).

### Treatment, clinical course and outcome

**Fig. 2:**
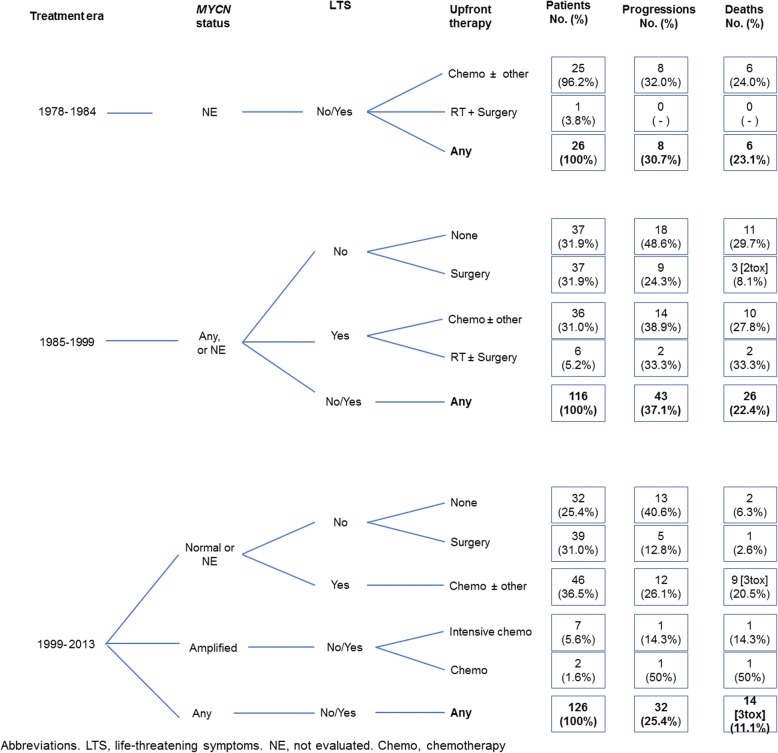
Progression-free and overall survival in 268 patients, by treatment era (**a** and **b**) and major sumptoms at diagnosis (**c** and **d**)

Details of clinical course and outcome in the 3 patient groups are reported in Fig. 2.

#### First treatment era (1979–1984)

**Fig. 3:**
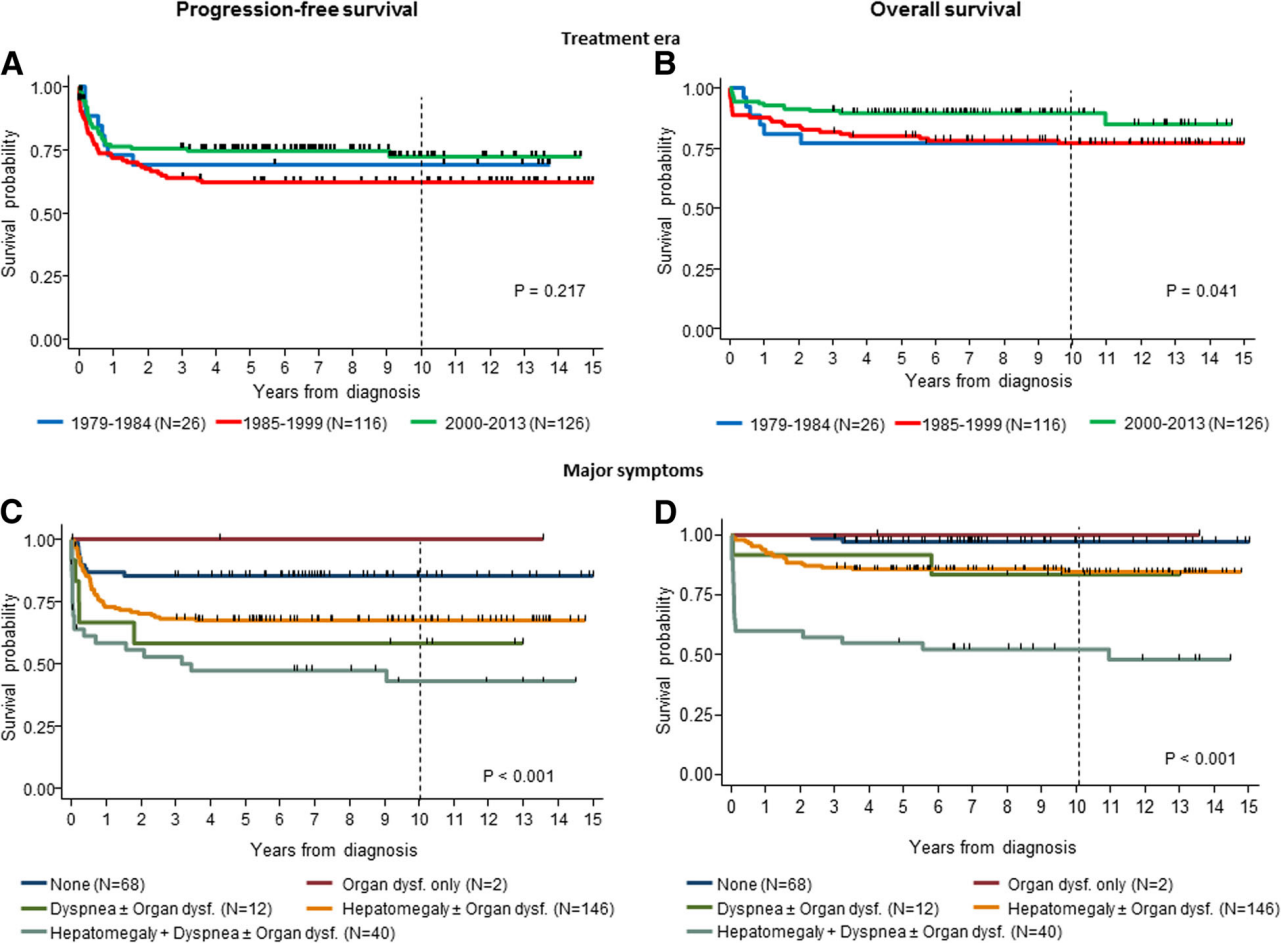
Progression-free and overall survival in 268 patients, by treatment era and major symptoms on diagnosis

Twenty-five/26 patients (96.2%) received upfront chemotherapy. One patient underwent hepatic irradiation plus primary resection. Eight patients (all treated with chemotherapy) showed disease progression 2–18 months (median, 7) after diagnosis, yielding a 10-year PFS of 69.2% (95% CI, 47.8–83.3). Six patients died at 4–24 months (median, 9), yielding a 10-year OS of 76.9% (95% CI, 55.7–88.9) (Fig. 3, plot A and B).

#### Second treatment era (1985–1999)

Of 116 patients, 74 (63.8%) presented without LTS. The wait-and-see approach was adopted in 37 (31.9%), 18 of whom developed disease progression; 11 of these died, including 3 with *MYCN* gene amplification. The other 37 patients (31.9%) underwent resection of the primary as the only therapy: 2 died of surgery-related complications, and 9 developed disease progression, one of whom died (Fig. 2). A silastic patch to allow abdominal enlargement was positioned in 3 patients, and was successful in two.

The remaining 42 patients (36.2%) presented with LTS. Thirty-six (31.0%) underwent upfront chemotherapy (plus primary resection in 8), 17 of whom developed disease progression (11 died); the remaining 6 (5.2%) were treated with hepatic irradiation (plus resection of the primary in one); 4 of the 6 showed disease progression and 3 died (Fig. 2). Three/42 patients (7.1%) with amplified *MYCN,* who were treated with chemotherapy (*n* = 1) or tumor resection (*n* = 2), are alive.

Overall, disease progression occurred in 43 patients 0–43 months (median, 3) after diagnosis, yielding an estimated 10-year PFS of 62.3% (95% CI, 52.7–70.5) (Fig. 3, plot A). A total of 26 deaths occurred after 0–115 months (median, 4), including 2 surgery-related, yielding a 10-year OS of 77.2% (95% CI, 68.3–83.9) (Fig. 3, plot B).

#### Third treatment era (2000–2013)

Of 126 patients, 9 (7.1%) had *MYCN* gene amplification. Of the 117 (93.4%) without *MYCN* amplification, 71 (56.3%) had no LTS on diagnosis; 32 (25.4%) of these underwent observation; disease progression ensued in 13 patients, 2 of whom died. The remaining 39 (31.0%) underwent primary resection, which was followed by disease progression in 5 cases (1 died). The 46 patients (30.9%) with LTS received upfront chemotherapy (plus tumor resection in 7); 3 died of chemotherapy-related complications, and 12 suffered disease progression, 6 of whom died (total: 9 deaths). Of the 9 patients with amplified *MYCN* gene, two received standard chemotherapy (with one fatal progression), and 7 intensive chemotherapy (with one fatal progression) (Fig. 2).

In summary, 32 patients suffered progression, yielding a 10-year PFS of 74.9% (95% CI, 66.2–81.6) (Fig. 3, plot A) and 11 patients died. Another three chemotherapy-related deaths occurred, bringing the overall death count to 14 (10-year OS = 89.7%; 95% CI, 82.9–93-9) (Fig. 3, plot B).

### Patient outcome and prognostic factors

Table 2 reports the 10-year PFS and OS for the entire study population, by era and clinical and biologic risk factors. PFS was 68.2% (95% CI, 62.1–73.5) in the whole cohort, without significant differences among the 3 eras, while OS was 82.7% (95% CI, 77.4–86.8) in the entire cohort and was better in the third era (89.7%; (95% CI, 82.9–93.9) than in the previous two (76.9 and 77.2%, respectively) (*P* = .041, test for trend) (Fig. 2, plot A and B). Gender did not influence PFS, while OS was better in females (88.5 vs 78.5%; *P* = 0.018). Patients diagnosed in the first month of life (0–29 days) did worse than those diagnosed subsequently (OS, 73.0% vs 85.5% *P* = 0.006). When survival estimates were stratified by month of diagnosis (Fig. 2) the differences among groups were not significant (*P* = 0.067, test for trend), with patients diagnosed in the 2nd, 3rd and 4th months of life showing similar “intermediate” outcomes, and those diagnosed after the 4th month having a better outcome (Fig. 1, plot B).

**Table 2:** PFS and OS by risk factors of 268 stage 4 s neuroblastoma patients

Entire cohort		Progressions	10-yrs PFS	*p*	Deaths	10-year OS	*p*
	No. (%)	No. (%)	% (95% CI)		No. (%)	% (95% CI)	
	268 (100)	83 (31)	68.2 (62.1–73.5)		46 (17.2)	82.7 (77.4–86.8)	
Treatment era							
1979–1984	26 (9.7)	8 (30.8)	69.2 (47.8–83.3)	0.217#	6 (23.1)	76.9 (55.7–88.9)	0.041#
1985–1999	116 (43.3)	43 (37.1)	62.3 (52.7–70.5)		26 (22.4)	77.2 (68.3–83.9)	
2000–2013	126 (47)	32 (25.4)	72.4 (62.4–80.1)		14 (11.1)	89.7 (82.9–93.9)	
Gender							
Male	156 (58.2)	52 (33.3)	65.4 (57.1–72.5)	0.24	34 (21.8)	78.5 (71.1–84.2)	0.018
Female	112 (41.8)	31 (27.7)	72.1 (62.7–79.5)		12 (10.7)	88.5 (80.3–93.4)	
Age, days							
0–29	60 (22.4)	23 (38.3)	59.8 (45.7–71.4)	0.033#	17 (28.3)	73.0 (59.7–82.5)	0.067#
30–59	40 (14.9)	14 (35.0)	65.0 (48.2–77.6)		8 (20.0)	80.0 (64.0–89.5)	
60–89	37 (13.8)	9 (24.3)	75.0 (57.5–86.1)		6 (16.2)	83.8 (67.4–92.4)	
90–119	40 (14.9)	15 (18.1)	61.6 (44.6–74.8)		7 (17.5)	81.0 (63.5–90.6)	
≥ 120	91 (33.9)	22 (26.5)	75.5 (65.2–83.1)		8 (8.8)	91.0 (82.7–95.4)	
Age, days							
0–29	60 (22.4)	23 (38.3)	59.8 (45.7–71.4)	0.058	17 (28.3)	73.0 (59.7–82.5)	0.006
30–365	208 (77.6)	60 (28.8)	70.7 (64.0–76.4)		29 (19.9)	85.5 (79.6–89.7)	
Symptoms at presentation							
None	17 (6.3)	1 (5.9)	94.1 (65.0–99.2)	< 0.001#	0	100	< 0.001#
Yes, minor	51 (19.0)	9 (17.7)	82.4 (68.8–90.4)		2 (3.9)	96.0 (84.9–99.0)	
Yes, major	200 (74.6)	73 (36.5)	62.4 (55.1–68.8)		44 (22.0)	77.9 (71.3–83.2)	
Hepatomegaly							
None	82 (30.6)	15 (18.3)	81.7 (71.5–88.5)	0.004	4 (4.9)	94.7 (86.5–98.0)	< 0.001
Yes	186 (69.4)	68 (36.6)	62.2 (54.6–68.9)		42 (22.6)	77.4 (70.6–82.9)	
Dyspnea							
None	216 (80.6)	57 (26.4)	73.4 (66.9–78.8)	< 0.001	24 (11.1)	88.4 (83.1–92.2)	< 0.001
Yes	52 (19.4)	26 (50)	47.1 (32.5–60.3)		22 (42.3)	59.5 (44.9–71.4)	
Organ dysfunctions							
None	234 (87.3)	72 (30.8)	68.8 (62.4–74.4)	0.714	36 (15.4)	84.0 (78.4–88.2)	0.032
Yes	34 (12.7)	11 (32.4)	64.5 (44.3–78.9)		10 (29.4)	73.5 (55.3–85.3)	
Combinations of major symptoms							
No major symptoms	68 (25.4)	10 (14.7)	85.3 (74.4–91.8)	< 0.001	2 (2.9)	97.0 (88.6–99.2)	< 0.001
Hepatomegaly ± Organ dysfunction	146 (54.5)	47 (32.2)	67.4 (59.0–74.4)		22 (15.1)	84.4 (77.2–89.5)	
Dyspnea ± Organ dysfunction	12 (4.5)	5 (41.7)	58.3 (27.0–80.1)		2 (16.7)	83.3 (48.2–95.6)	
Hepatomegaly + Dyspnea (±Organ dysfunction)	40 (14.9)	21 (52.5)	43.0 (26.3–58.7)		20 (50)	52.4 (36.0–66.4)	
Organ dysfunction only	2 (0.8)	0	100		0	100	
Abdominal mass							
None	234 (87.3)	77 (32.9)	66.1 (59.5–71.9)	0.078	45 (19.2)	80.6 (74.7–85.2)	0.023
Yes	34 (12.7)	6 (17.6)	82.4 (64.9–91.7)		1 (2.9)	97.1 (80.9–99.6)	
Cervical mass							
None	257 (95.9)	80 (31.1)	68.0 (61.8–73.4)	0.797	44 (17.1)	82.8 (77.5–87.0)	0.896
Yes	11 (4.1)	3 (27.3)	72.7 (37.1–90.3)		2 (18.2)	77.9 (35.4–94.2)	
Skin nodules							
No	226 (84.3)	64 (28.3)	70.7 (64.1–76.3)	0.029	36 (15.9)	83.9 (78.2–88.3)	0.255
Yes	42 (15.7)	19 (45.2)	54.8 (38.7–68.3)		10 (23.8)	75.9 (59.7–86.2)	
Neurologic symptoms							
None	256 (95.5)	77 (30.1)	69.1 (62.9–74.5)	0.148	45 (17.6)	82.6 (77.3–86.8)	0.404
Yes	12 (4.5)	6 (50)	50.0 (20.9–73.6)		1 (8.3)	75.0 (12.8–96.1)	
Primary site							
Adrenal	175 (65.3)	59 (33.7)	65.8 (58.2–72.3)	0.609	33 (18.8)	80.4 (73.5–85.8)	0.489
Abdomen	49 (18.3)	11 (22.5)	75.0 (58.7–85.6)		7 (14.3)	87.8 (74.8–94.3)	
Thorax	22 (8.2)	7 (31.8)	68.2 (44.6–83.4)		1 (4.5)	95.5 (71.9–99.4)	
Neck	9 (3.4)	3 (33.3)	66.7 (28.2–87.8)		2 (22.2)	71.1 (23.3–92.3)	
Not detected	13 (4.9)	3 (23.1)	76.9 (44.2–91.9)		3 (23.1)	76.9 (44.2–91.9)	
Primary site thorax							
No	246 (91.8)	76 (30.9)	68.9 (62.7–74.4)	0.944	45 (18.3)	81.5 (75.8–85.9)	0.112
Yes	22 (8.2)	7 (31.8)	68.2 (44.6–83.4)		1 (4.5)	95.5 (71.9–99.4)	
Liver infiltration							
No	38 (14.2)	7 (18.4)	81.6 (65.2–90.8)	0.084	0	100	0.004
Yes	230 (85.8)	76 (33)	65.9 (59.3–71.8)		46 (20)	79.8 (73.7–84.5)	
Positive bone marrow cytology							
No	157 (58.6)	49 (31.2)	68.3 (60.3–75.0)	0.753	28 (17.8)	81.9 (74.9–87.2)	0.643
Yes	111 (41.4)	34 (30.6)	68.1 (58.2–76.2)		18 (16.2)	83.6 (74.6–89.6)	
Urine VMA (222 tested)							
Normal	59 (26.6)	16 (27.1)	72.4 (59.0–82.1)	0.381	5 (8.5)	91.5 (80.8–96.4)	0.13
Elevated	163 (73.4)	53 (32.5)	66.7 (58.7–73.5)		27 (16.6)	83.9 (77.2–88.7)	
Urine HVA (112 tested)							
Normal	18 (16.1)	4 (22.2)	77.8 (51.1–91.0)	0.253	2 (11.1)	88.9 (62.4–97.1)	0.846
Elevated	94 (83.9)	33 (35.1)	64.0 (53.0–73.0)		12 (12.8)	88.3 (79.9–93.3)	
Serum LDH (227 tested)							
Normal	131 (57.7)	33 (25.2)	74.6 (66.2–81.2)	0.055	14 (10.7)	89.1 (82.3–93.4)	0.031
Elevated	96 (42.3)	35 (36.5)	60.9 (49.6–70.4)		20 (20.8)	78.8 (68.1–86.2)	
Serum Ferritin (193 tested)							
Normal	116 (60.1)	34 (29.3)	70.5 (61.2–77.9)	0.859	15 (12.9)	87.0 (79.4–92.0)	0.425
Elevated	77 (39.9)	22 (28.6)	70.0 (58.1–79.1)		13 (16.9)	82.8 (72.1–89.6)	
*MYCN* gene (183 tested)							
Normal	168 (91.8)	53 (31.5)	67.1 (59.1–73.9)	0.673	21 (12.5)	86.8 (79.9–91.5)	0.021
Amplified	15 (8.2)	6 (40)	60.0 (31.8–79.7)		5 (33.3)	66.7 (37.5–84.6)	
1p chromosome *(138 tested)*							
Normal	110 (79.7)	31 (28.2)	71.3 (61.7–78.8)	0.141	10 (9.1)	89.1 (79.2–94.4)	< 0.001
Deleted	28 (20.3)	13 (46.4)	50.8 (29.7–68.5)		10 (35.7)	67.9 (47.3–81.8)	
DNA index *(121 tested)*							
Aneuploid	80 (66.1)	24 (30)	70.0 (58.7–78.8)	0.734	3 (3.8)	96.3 (88.8–98.8)	< 0.001
Di-tetraploid	41 (33.9)	14 (34.2)	65.8 (49.1–78.1)		10 (24.4)	70.8 (49.2–84.5)	
Histology INPC (75 tested)							
Favourable	69 (92)	16 (23.2)	73.9 (59.3–83.9)	0.78	5 (7.3)	94.2 (85.3–97.8)	0.147
Unfavourable	6 (8)	2 (33.3)	66.7 (19.5–90.4)		2 (33.3)	66.7 (19.5–90.4)	
Upfront treatment							
Observation	69 (25.8)	31 (44.9)	55.1 (42.6–65.9)	0.015	13 (18.8)	81.1 (69.7–88.6)	< 0.001
Chemotherapy	90 (33.6)	29 (32.2)	66.8 (55.8–75.6)		23 (25.6)	73.2 (62.2–81.5)	
Resection of primary	76 (28.4)	14 (18.4)	81.1 (70.2–88.3)		4 (5.3)	94.3 (85.4–97.9)	
Chemotherapy + Resection of primary	19 (7.1)	4 (21.1)	76.6 (48.0–90.7)		2 (10.5)	94.7 (68.1–99.2)	
Radiotherapy + Other	14 (5.2)	5 (35.7)	64.3 (34.3–83.3)		4 (28.6)	71.4 (40.6–88.2)	

Abbreviations. PFS progression-free survival, OS overall survival, VMA vanillylmandelic acid, HVA homovanillic acid, LDH lactate dehydrogenase, INPC international neuroblastoma pathology classification

#; test for trend

The presence of major symptoms on diagnosis significantly affected PFS and OS. The combination of hepatomegaly and dyspnea +/− organ dysfunction was associated with the lowest PFS and OS (43.0 and 52.4%, respectively) (Table 2 and Fig. 3, plot C and D). A significant association with better OS, but not better PFS, was found in the case of an abdominal mass in the absence of hepatomegaly (OS, 97.1% vs 80.6%; *P* = 0.023), absence of liver infiltration (OS, 100% vs 79.8%; *P* = 0.004), normal levels of serum LDH (OS, 89.1% vs 78.8%; *P* = 0.031), and absence of abnormalities of biologic features, in particular *MYCN* gene (OS, 86.8% vs 66.7%; *P* = 0.021), 1p chromosome (OS, 89.1% vs 67.9%; *P* < 0.001) and DNA index (OS, 96.3% vs 70.8%; *P* < 0.001) (Table 2).

Patients who underwent early resection of the primary tumor, either alone or combined with chemotherapy, had a more favorable outcome (PFS, 81.1 and 76.6%; OS, 94.3 and 94.7%, respectively) than those who were initially observed (PFS 55.1%; OS, 81.1%), those who received upfront chemotherapy (PFS, 66.8%; OS, 73.2%), and those who were treated with liver irradiation, alone or with other modalities (PFS, 64,3%; OS, 71.4%) (*P* < 0.001) (Table 2).

Multivariable analysis of the combined effect of the different risk factors was limited to evaluation of the clinical and demographic data significantly associated with outcome in the univariate analysis (Table 3). It was therefore carried out in 266/268 patients, as 2 who had organ dysfunction as the only major symptom had no events, and thus were not suitable for inclusion in the model. The only factor that independently affected the risk of disease progression and/or death was the presence of major symptoms. Compared to subjects without major symptoms, those who had the combination of hepatomegaly and dyspnea ± organ dysfunction had a 5.5-fold higher risk of progression (95% CI, 2.6–11.8) and a 24.1-fold higher risk of death (95% CI, 5.6–103.4) (Table 3). Patients with hepatomegaly ± organ dysfunction and those with dyspnea ± organ dysfunction had 3.1 (95% CI, 1.1–9.3) and 2.2 (95% CI, 1.1–4.3) -fold higher risks of progression and 4.6 (95% CI, 0.6–33.2) and 4.6 (95% CI, 1.1–19.8) -fold higher risk of death, respectively, than those without major symptoms (Table 3).

**Table 3:** Multivariable analysis in 266* patients with stage 4 s neuroblastoma

	No. (%) 266	PFS			*p*	OS			
		Univariate	*p*	Multivariate		Univariate		Multivariate	
		HR (95% CI)		HR (95% CI)		HR (95% CI)	*p*	HR (95% CI)	*p*
Major symptoms									
None	68 (25.4)	1	< 0.001	1	< 0.001	1	< 0.001	1	< 0.001
Dyspnea ± Organ dysfunction	12 (4.5)	3.5 (1.2–10.2)		3.1 (1.1–9.3)		5.9 (0.8–41.6)		4.6 (0.6–33.2)	
Hepatomegaly ± Organ dysfunction	146 (54.5)	2.4 (1.2–4.7)		2.2 (1.1–4.3)		5.3 (1.2–22.6)		4.6 (1.1–19.8)	
Hepatomegaly + Dyspnea (± Organ dysfunction)	40 (14.9)	5.5 (2.6–11.7)		5.5 (2.6–11.8)		24.4 (5.7–104.4)		24.1 (5.6–103.4)	
Treatment era									
1979–1984	25 (9.4)	1	0.202#	1	0.355#	1	0.041#	1	0.049#
1985–1999	116 (43.6)	1.3 (0.6–2.7)		1.4 (0.7–3.0)		1 (0.4–2.4)		1.2 (0.5–2.8)	
2000–2013	125 (47.0)	0.8 (0.4–1.8)		0.9 (0.4–2.0)		0.5 (0.2–1.3)		0.5 (0.2–1.3)	
Gender									
Male	154 (57.9)	1	0.209	1	0.707	1	0.013	1	0.091
Female	112 (42.1)	0.8 (0.5–1.2)		0.8 (0.5–1.3)		0.5 (0.2–0.9)		0.6 (0.3–1.1)	
Age, days									
0–29	60 (22.6)	1	0.077	1	0.637	1	0.011	1	0.639
30–365	206 (77.4)	0.6 (0.4–1.0)		0.9 (0.5–1.5)		0.4 (0.2–0.8)		0.9 (0.4–1.6)	
Abdominal mass									
None	232 (87.2)	1	0.052	1	0.751	1	0.006	1	0.813
Yes	34 (12.8)	0.5 (0.2–1.1)		1.2 (0.4–3.6)		0.1 (0.0–1.0)		0.7 (0.1–8.4)	

Abbreviations. PFS progression-free survival, OS overall survival, HR hazard ratio, CI confidence interval

*, excluding 2 patients presenting with organ dysfunction as only major symptom

#, test for trend

## Discussion

Overall, we found few significant differences in the presenting features of patients diagnosed in the successive periods, the main one regarding the number of patients who presented without symptoms; this was chiefly because of the increasing use of ultrasound in pregnancy and early life.

As in one published series [12], but not in others [11, 13], male gender prevailed. Females, however, had a significantly better outcome, although this previously unreported finding was not confirmed on multivariate analysis. Our data confirm the worse outcome of patients diagnosed in the first 2 months. However, the highest number of deaths occurred in the first month of life, while comparable numbers of deaths occurred among those diagnosed in the 2nd, 3rd, and 4th months.

The presence of any major symptom was associated with lower OS (77.4% for hepatomegaly, 59.5% for dyspnea; 73.5% for organ dysfunctions). However, it was the association of hepatomegaly and dyspnea that drastically lowered OS to 52.4%; this was confirmed on multivariate analysis. Patients without major symptoms usually presented in good condition and did well (OS, 96.0%). The absence of symptoms in the 17 patients whose disease was discovered by means of ultrasound was associated with a 100% OS.

The commonest primary tumor site was adrenal, and bilateral involvement was observed in 9 cases. The high frequency of bilateral involvement (5.1% vs 0.2% in the entire RINB population; unpublished) has previously been reported [12–14], and been considered an expression of the multifocal character of stage 4 s disease [28]. Retroperitoneal ganglia were four times less likely to be the primary site. In these instances, the tumor mass, by definition, crossed the midline, and this would have excluded these patients from enrollment as stage 4 s. However, the fact that similar patients were included in other series [10, 11, 13], and that their outcome was comparable to that of our patients with adrenal primary tumors (87.8% vs 80.4%; not significant) justifies their inclusion. On the other hand, the concept of midline-crossing no longer appears in the recent INRG (International Neuroblastoma Risk Group) definition [3].

Abnormal biologic features did influence patient outcome. *MYCN* gene amplification was found in 8.2% of the 183 patients tested, a lower figure than in infants with stage 4 disease and older patients [14, 18, 22]. Although the 86.8% OS of patients with a normal *MYCN* gene was significantly better than the 66.7% OS of patients with an amplified gene (*P* = .021), 10 of 15 patients with abnormal *MYCN* survived, including 3 of the 6 who received standard chemotherapy or underwent primary resection as the only therapy. Similar results have previously been reported by other investigators, who have hypothesized that the biology of some *MYCN*-amplified favorable tumors differs from that of advanced-stage tumors [29, 30]. Patients with amplified *MYCN* may have gained some advantage from an aggressive therapeutic approach, as only one of the 7 so treated died of disease. This supports the data of a recent SIOPEN study [27]. Both abnormalities of 1p chromosome and a di/tetraploid DNA index were associated with worse OS (67.9% vs 89.1; *P* < .001 and 70.8% vs 96.3%; *P* = .001), confirming previous data [11–14].

The influence of therapeutic modalities on outcome was not easily assessable. Patients assigned to a wait-and-see policy were free from major symptoms on presentation. Nevertheless, their survival was no better than that of the overall population (81.1% vs 81.5%); indeed, 45% of them eventually suffered disease progression and 19% died. Whether administering upfront chemotherapy to these patients would have reduced the number of progressions and deaths remains unclear. With the exception of the first era, it was the presence of major symptoms on presentation and/or the evidence of rapid disease progression that led clinicians to initiate chemotherapy. This was not always life-saving, as these patients eventually had a low survival probability (73.2%).

Overall, the chance of cure for our stage 4 s neuroblastoma patients did improve over time, reaching a survival probability of 89.7%, which is close to the rates reported in recent series [13, 14]. The OS of patients of the first 2 eras was very close (76.9% vs 77.2%). However, it should be noted that no fatal progression was recorded in patients of the first treatment era, suggesting that, in the initial years of the study, some critical patients might have succumbed without reaching oncologic attention. Patients of the third treatment era did better. The following reasons may partially account for this result. First, the majority of asymptomatic patients (all of whom survived) belonged to this group. Second, the prevalence of hepatomegaly was lower and that of abdominal tumors was higher in later patients, both of which are features associated with a favorable outcome. Third, patients with amplified *MYCN* gene did better when they underwent aggressive therapy, which was administered to later patients only. Finally, enrollment of the third era patients in a large international SIOPEN study may have meant that they underwent a better management strategy.

Patients who underwent early primary tumor resection, either as the only therapy or in association with chemotherapy, did very well (OS, 94.3 and 94.7%, respectively), supporting the hypothesis that primary resection is associated with favorable outcome [31, 32]. However, as patients undergoing early primary tumor resection usually presented in good condition, their outcome did not come as a surprise. Indeed, with the exception of the 2 surgery-related deaths, which occurred in the middle years of the study, operations were usually performed safely. Whether resection of the primary tumor may confer a real survival advantage remains a matter of debate [26]. Patients in whom radiotherapy was part of the treatment did poorly, as it was usually undertaken in severely ill patients (OS, 71.4%).

## Conclusions

Raising the cure rate above the currently achievable 90% is a challenge for pediatric oncologists. The main obstacle to full patient cure is constituted by the association of hepatomegaly and dyspnea. In these patients, symptom progression can be overwhelmingly rapid and frustrate “traditional” therapy. Saving these patients could possibly depend on the timely use of surgical techniques that require specific operator experience. The positioning of a silastic patch in the case of life-threatening abdominal expansion is an established procedure [33, 34]. Intra-arterial liver chemoembolization has recently been attempted with success in infants who fail to respond to chemotherapy [35, 36]. Finally, liver transplant has proved life-saving in some patients [37, 38]. A sequential treatment algorithm based on initial tumor behavior and response to therapy has been proposed by Weintraub et al.[39] According to this, chemotherapy should be reserved for patients who present with, or develop, a rapid increase in abdominal girth, especially when this is associated to respiratory distress. Non-responders should be considered for immediate liver chemoembolization. Liver transplantation could be undertaken in the event of failure, but must be carried out in the few institutions with specific expertise.

The establishment of a well-organized network of centers that deal with high-risk neuroblastoma patients is a prerequisite to the implementation of such a strategy. Identifying these centers through the European Reference Networks of the European Commission (ec.europa.eu/health/ern_en) is an important step in this direction. Stage 4 s patients with risk features should be identified early and, in the event of poor response to initial therapy, promptly referred to a dedicated institution.

### Additional file

Additional file 1: **Table S1.** Outlines of therapy for stage 4 s neuroblastoma patients (DOCX 16 kb)

## Appendix

*The following Italian institutions participated in this study (with main investigators):*

IRCSS Istituto Giannina Gaslini, Genova (Bruno De Bernardi, Riccardo Haupt, Alberto Garaventa, Anna Rita Gigliotti, Stefano Avanzini, Claudio Granata, Angela Rita Sementa, Katia Mazzocco); Bambino Gesù Children’s Hospital, Roma (Aurora Castellano, Alessandro Inserra); Department of Pediatric Hematology-Oncology, University Hospital, Catania (Andrea Di Cataldo); Department of Woman and Children’s Health, University of Padova, Padova (Elisabetta Viscardi, Giovanni Cecchetto); Division of Pediatric Oncology, Istituto Nazionale Tumori, Milano (Marta Podda, Roberto Luksch); Hemato-Oncology Unit, S.Orsola Malpighi Policlinic, Bologna (Fraia Melchionda); Department of Pediatrics, University La Sapienza, Roma (Alessandra de Grazia, Anna Clerico); Department of Pediatrics, University of Palermo, Palermo (Paolo D’Angelo, Fortunato Siracusa); Department of Hematology-Oncology, Regina Margherita Children’s Hospital, Torino (Elisa Tirtei, Maurizio Bianchi); Oncology Unit, Burlo Garofalo Children’s Hospital, Trieste (Andrea Giulio Zanazzo, Marco Rabusin); Paediatric Surgery Unit, Fondazione IRCCS Ca′ Granda Ospedale Maggiore Policlinico, Milano (Anna Maria Fagnani); Santobono-Pausilipon Children’s Hospital, Napoli (Simona Vetrella); Department of Pediatrics, Civic Hospital, Bergamo (Massimo Provenzi); Department of Pediatrics, University Hospital, Bari (Francesco De Leonardis); Department of Pediatrics, Civic Hospital, Brescia (Carmelita D’Ippolito, Fabian Schumacher); Anna Meyer Children’s Hospital, Firenze (Annalisa Tondo, Angela Tamburini); Department of Pediatric Haematology-Oncology, Agostino Gemelli Hospital, Roma (Stefano Mastrangelo); Pediatric Microcitemic Hospital, Cagliari (Antonella Nonnis, Rosa Maria Mura); Pediatric Onco-Hematology, University HospitalVerona (Simone Cesaro); Paediatric Hematology-Oncology Unit, University Policlinic, Modena (Monica Cellini); Pediatric Onco-Hematology, Civic Hospital, Parma (Patrizia Bertolini); G Salesi Children’s Hospital, Ancona (Paolo Pierani); Pediatric Oncology Unit, Second University Hospital, Napoli (Elvira Pota, Fiorina Casale); Pediatric Hematology-Oncology, Casa Sollievo della Sofferenza Hospital, S. Giovanni Rotondo, (Lucia Miglionico); Pediatric Hematology-Oncology, S. Matteo University Hospital, Pavia (Federico Bonetti); Paediatric Hematology-Oncology, University Hospital, Pisa (Emanuela De Marco); Pediatric Hematology-Oncology, Civic Hospital, Ferrara (Roberta Burnelli, Simona Rinieri).

## Abbreviations

AIEOP: Italian Association of Pediatric Hematology-Oncology
INPC: International Neuroblastoma Pathology Classification
INRG: International Neuroblastoma Risk Group
INRGSS: International Neuroblastoma Risk Group Staging System
INSS: International Neuroblastoma Staging System
RINB: Italian Neuroblastoma Registry
SIOPEN: International Society of Pediatric Oncology Europe Neuroblastoma
