# Supplementary material for: Stage 4 s neuroblastoma: features, management and outcome of 268 cases from the Italian Neuroblastoma Registry
Source: Ital J Pediatr. 2019 Jan 11;45:8. doi: 10.1186/s13052-018-0599-1 (PMC6329141; doi:10.1186/s13052-018-0599-1)
Supplement: Supplementary file 1 — Table S1. Outlines of therapy for stage 4 s neuroblastoma patients (DOCX 16 kb) [file 13052_2018_599_MOESM1_ESM.docx]

**SUPPLEMENTAL TABLE Outlines of therapy for stage 4s neuroblastoma patients**

| Treatment era | Age | LTS | *MYCN* gene | Chemotherapy | Primary tumor resection | Radiation therapy |
| --- | --- | --- | --- | --- | --- | --- |
| 1979-1984 | < 6 mos | No/Yes | NE | A) 2 PTC courses | Encouraged in absence of risks | Of hepatic area upon local decision |
|  | 6-11 mos | No/Yes | NE | As A) followed by 4-8 courses of various drug associations | idem |  |
| 1985-1999 | 0-11 mos | No | Normal or NE | No | idem | No |
|  | 0-11 mos | Yes | Normal or NE | 2-4 courses of various drug associations | idem |  |
| 2000-2013 | 0-11 mos | No | Normal or NE | No | idem | No |
|  | 0-11 mos | Yes | Normal or NE | 2-4 courses of Carbo/VP16 | idem | No |
|  | 0-11 mos | No/Yes | Amplified | Intensive chemotherapy | idem | Of primary site after surgery |

**Abbreviations**.

LTS, life-threatening symptoms. NE, not evaluated. PTC, Peptichemio. Carbo, carboplatin. VP16, etoposide.
